# O^6^-methylguanine DNA methyltransferase (MGMT) expression in U1242 glioblastoma cells enhances *in vitro* clonogenicity, tumor implantation *in vivo*, and sensitivity to alisertib-carboplatin combination treatment

**DOI:** 10.3389/fncel.2025.1552015

**Published:** 2025-04-22

**Authors:** Müge Sak, Brian J. Williams, Andrew J. Hey, Mayur Sharma, Leslie Schier, Megan J. Wilson, Mahatma Ortega, Alyssa I. Lara, Mikaela N. Brentlinger, Norman L. Lehman

**Affiliations:** ^1^Departments of Biochemistry and Molecular Genetics, University of Louisville, Louisville, KY, United States; ^2^Pathology and Laboratory Medicine, University of Louisville, Louisville, KY, United States; ^3^Neurological Surgery, University of Louisville, Louisville, KY, United States; ^4^Brown Cancer Center, University of Louisville, Louisville, KY, United States; ^5^Departments of Pathology and Laboratory Medicine, Baylor Scott & White Health, Baylor College of Medicine, Temple, TX, United States; ^6^Department of Pathology and Immunology, Baylor College of Medicine, Houston, TX, United States

**Keywords:** Aurora A Kinase, AURKA, *O*^6^-methylguanine DNA methyltransferase, MGMT, GBM, anchorage-independent growth, clonogenicity, *in vivo*

## Abstract

Glioblastoma (GBM) is the most common and aggressive primary adult CNS tumor. Increased understanding of glioma biology is needed for novel treatment strategies and maximization of current therapies. The action of the widely used antiglioma drug, temozolomide (TMZ), relies on its ability to methylate DNA guanine bases leading to DNA double strand breaks and apoptosis. However, glioma cells capable of reversing guanine methylation via the repair enzyme *O*^6^-methylguanine DNA methyltransferase (MGMT) are resistant to TMZ. GBMs exhibiting high MGMT expression, reflected by MGMT gene promoter hypomethylation, respond poorly to both chemo- and radiation therapy. To investigate possible non-canonical biological effects of MGMT and develop a tool to investigate drug sensitivity and resistance, we generated MGMT knockout (KO) U1242 GBM cells. MGMT KO U1242 cells showed substantially increased sensitivity to TMZ *in vivo*, and unlike wildtype U1242 cells, failed to form tumors in nude mouse brains. They also showed reduced growth in soft agar, as did wildtype U1242 and additional glioma cell lines in which MGMT expression was knocked down by siRNA. MGMT thus possesses cellular functions related to tumor cell engraftment and anchorage-independent growth beyond guanine methyltransferase repair. We additionally show that the combination of the AURKA inhibitor alisertib and carboplatin selectively induces apoptosis in high MGMT expressing wildtype U1242 cells versus MGMT KO U1242 cells and extends survival of mice orthotopically implanted with wildtype U1242 cells. This or other platinum-based drug combinations may represent a potentially effective treatment approach to chemotherapy for GBM with MGMT promoter hypomethylation.

## Introduction

Glioblastoma (GBM) is the most common and aggressive central nervous system tumor in adults and is defined, in part, as an isocitrate dehydrogenase 1 and 2 wildtype, WHO grade 4 glioma (Stoyanov et al., 2022). The average survival of GBM patients is 10–15 months from the time of diagnosis (Cohen et al., 2013). Improved understanding of the biology of GBM is critical for the discovery of novel and more effective therapies. Effective chemotherapeutic approaches may require a combination of agents (Jiang et al., 2014).

*O*^6^-methylguanine DNA methyltransferase (MGMT) is a DNA repair enzyme that reverses damaging methylation of guanine residues in DNA caused by alkylating agents. MGMT transfers these *O*^6^-methyl groups from guanines to its own cysteine 145, resulting in its degradation (Okamoto et al., 2002). High MGMT expression in tumors predicts a poor response to alkylating agent chemotherapies such as the commonly used GBM chemotherapy drug temozolomide (TMZ). This is because tumors with high MGMT expression are able to repair the methylation damage caused by TMZ.

Epigenetic hypermethylation of CpG islands within the MGMT gene promoter and untranslated regions of its first exon suppresses MGMT gene expression. This regulation is exploited clinically to indirectly predict MGMT protein expression in patient glioma samples in order to gauge patient response to TMZ or other alkylating agents. MGMT promoter methylation status is determined in routine formalin-fixed, paraffin-embedded glioma tumor specimens, usually by bisulfide treatment of tumor DNA followed by DNA sequencing (Weller et al., 2010). GBM with hypomethylated MGMT promoters, and thus resistant to TMZ are most common and tend to be the most difficult-to-treat primary adult brain tumors. Over 50% of GBM cases have hypomethylated MGMT promoters and high MGMT expression (Brandes et al., 2008). Patient survival is significantly less when the MGMT promoter is hypomethylated (Hegi et al., 2008). In a WHO 2021 cohort, the survival of GBM patients with or without MGMT promoter methylation was significantly different (*p* = 0.0001) with a mean survival of 478 days for patients with MGMT promoter methylated tumors versus 142 days for patients with MGMT promoter unmethylated tumors (Stoyanov et al., 2022).

Here we used cell culture and an orthotopic human xenograft mouse model to investigate whether MGMT KO affected cellular proliferation, clonogenicity in soft agar and tumor growth *in vivo*. We previously found evidence of synergistic growth inhibitory and apoptosis-inducing cytotoxic effect of alisertib and carboplatin combination treatment to be selective in high MGMT-expressing GBM cell lines (Sak et al., 2019). Using MGMT KO and exogenous MGMT rescue we confirmed that this effect is directly related to the presence of MGMT expression.

## Materials and methods

### Cell lines

Experiments were performed with T98, LN18, U87 (American Type Culture Collection), and U1242 human GBM cell lines. STR profiling of U1242 cells was performed at the University of Arizona Genetics Core for authentication.

### Drugs and vehicles

Alisertib was purchased from Selleckchem, dissolved in DMSO, and further diluted in water for *in vitro* experiments. For *in vivo* experiments, alisertib was obtained from the National Cancer Institute’s Cancer Therapy Evaluation Program (CTEP) and dissolved in water consisting of 10% (2-hydroxypropyl)-β-cyclodextrin, 1% sodium bicarbonate. Carboplatin was purchased from Selleckchem and dissolved in water for *in vitro* experiments. For *in vivo* experiments, USP-grade carboplatin was purchased from Millipore Sigma and dissolved in 43% ethanol, 33% polypropylene glycol, 24% cremophor, and diluted 1:16 with sterile water.

### IDH1/2 mutational analysis

U1242 cell pellets were resuspended in RPMI 1640 and vortexed for 10 s with 300 μl of mineral oil, followed by heating at 80°C for 2 min, and cooling to room temperature. A solution containing 250 μl of lysis buffer, proteinase K, and blue dye was added to the samples, followed by vortexing and centrifugation at 10,000 × *g* for 20 s. After incubating at 56°C for 30 min and overnight at 70°C, 10 μl of RNase A solution was added and the samples were centrifuged at 10,000 × *g* for 5 min. The aqueous phase containing the DNA was purified using the QIAamp kit (QIAGEN) according to the manufacturer’s protocol. The DNA was quantified using a spectrophotometer. For PCR amplification, a master mix was prepared with primers designed to detect mutations in IDH1 (R132H, R132C, R132S, R132G, R132L, R132V, R100Q) and IDH2 (R172K, R172M, R172W, R172S, R172G). Twenty microliters of master mix was added to each well of a 72-well rotor, followed by 2 μl of DNA eluate or controls (Positive, Wild Type, or Water Control). Samples and controls were loaded onto the RotorGene thermocycler (QIAGEN), gain optimization settings adjusted, and the thermal cycling program was initiated.

### CRISPR/Cas9 knockout

Two separate guide RNAs (gRNAs) that target exon 2 of the MGMT gene are provided by the Origene MGMT Knockout Kit (KN201612). U1242 cells were seeded and transfected with gRNA (GGTGCGCACCGTTTGCGACT) using Turbofectin 8.0 (TF81001) transfection agent. A total of 48 h post-transfection, cells were passaged, and transfected cells were selected with 2 μg/ml puromycin. After puromycin selection, 1 cell/well was seeded in a 96-well plate for clonal selection. Single colonies were further extended in six-well plates and MGMT expression was screened via western blotting.

### Annexin V binding

Drug combination effects were tested by apoptosis assay via annexin V binding experiments. Drug-treated cells were stained with Alexa Fluor 594 annexin V conjugate (Thermo Fisher) and analyzed with a Countess II FL cell counter equipped with a Texas Red light cube (Thermo Fisher).

### Western blotting

Western blotting was performed using lysis buffer with the addition of 1 mM sodium orthovanadate and 5 mM sodium fluoride. Protein (10 μg) was run on 12% Bis-Tris gels (Thermo Fisher) and blotted onto PVDF membranes. MGMT (Invitrogen, 35-7000, 1:500) and β-actin (Sigma, A2228, 1:10,000) primary antibodies were incubated at 25°C for 90 min, and HRP-conjugated anti-mouse secondary antibodies (Cell Signaling; 1:2000) were incubated for 60 min at 25°C.

### MGMT overexpression

Cells were seeded in 6-well plates at 1 × 10^6 cells/well and transfected with 2.5 μg human MGMT-pCMV6 (NM_002412) (Origene RC229131) or empty pCMV6-Entry Myc-DDK-tagged vector (Origene PS100001) using OptiMEM and Lipofectamine 3000 (Invitrogen, Waltham, MA) for 72 h. Overexpression was confirmed by western blot.

### siRNA MGMT knockdown

Cells at approximately 60% confluence in 6-well plates were transfected with 25 nM ON-TARGETplus SMARTpool siRNA targeting human MGMT (Dharmacon, L-008856-01-0005) or ON-TARGETplus control non-targeting pool siRNA (Dharmacon, D-001810-10-05) in OptiMEM with DharmaFECT 1 for 48 h. MGMT knockdown was confirmed by western blot.

### Soft agar assays

A six-well tissue culture plate was coated with 1 ml 0.5% low melting temperature agar (15517-022, Invitrogen) for the base layer. After the base layer was solidified, 10,000 cells/well were mixed with 0.4% low melting temperature agar and added on top of the base layer. Following the solidification of the cell layer, 2 ml culture media was added. Cell colonies were photographed every 3 days via a Zeiss Axiocam camera system connected to an inverted microscope. Photomicrographs were uploaded to the NIH ImageJ software^1^ and colony sizes were measured and quantified using the measure tool in ImageJ software. Measurements for all observed colonies in each well were recorded for day 1, day 4, day 7, and day 10. Statistical significances were determined via GraphPad Prism using two-way ANOVA. Representative photomicrographs are shown in the figures.

### GBM mouse model

All animal experiments were approved by the University of Louisville IACUC (protocol 21867). Orthotopic xenograft implantation of human GBM cells into Foxn1nu athymic mice (Jackson Laboratory) was performed as previously described (Van Brocklyn et al., 2014). U1242 MGMT wildtype (U1242 MGMT WT) or MGMT knockout (U1242 MGMT KO) cells were injected into the right caudate nucleus of mouse brains using a stereotactic frame and microinjection unit (Kopf).

For MGMT WT versus MGMT KO U1242 cell *in vivo* tumor cell engraftment comparison, 15 female mice were used and each of five experimental groups had three mice that were implanted with MGMT WT or MGMT KO cells. For the alisertib + carboplatin treatment animal survival experiments, 28 mice (14 male and 14 female) were randomly assigned to treatment groups alternating male and female animals. Three animals failed to develop tumors. The number of animals per group with successful tumor engraftment was vehicle only, *n* = 5; alisertib only, *n* = 7; carboplatin only, *n* = 6; and alisertib + carboplatin, *n* = 7. Alisertib (20 mg/kg) or vehicle was administered orally by gavage twice per day, 5 days per week, beginning 5 days after tumor implantation, and continued for the duration of the experiment. Carboplatin (60 mg/kg) or vehicle was injected intraperitoneally once every 4 weeks, beginning 5 days after tumor implantation. Animals were euthanized by IACUC-approved endpoint criteria. Animal survival was assessed by Kaplan-Meier analysis using GraphPad Prism 9.2.0. H&E staining was performed for the visualization of tumors.

### Statistical analysis

GraphPad Prism 9.2.0 was used to perform ANOVA and Kaplan-Meier analyses. Data points in figures represent individual biological replicates and data is presented as the mean ± SD.

## Results

### MGMT knockout and knockdown significantly inhibits anchorage-independent growth of GBM cells

U1242 is a GBM cell line that forms aggressive invasive tumors when intracranially implanted in mice (Westermark et al., 1973; Zhao et al., 2010). The absence of IDH1 or IDH2 mutations is an essential characteristic of GBM as defined in the 2021 WHO classification of CNS tumors (Louis et al., 2021). IDH1/2 gene wildtype status was confirmed in U1242 cells by PCR as described in the methods section.

In this study we knocked-out (KO) the MGMT gene in U1242 GBM cells using CRSPR-Cas9 and also knocked-down (KD) MGMT mRNA expression in U1242 and additional glioma cell lines by siRNA.

**FIGURE 1:**
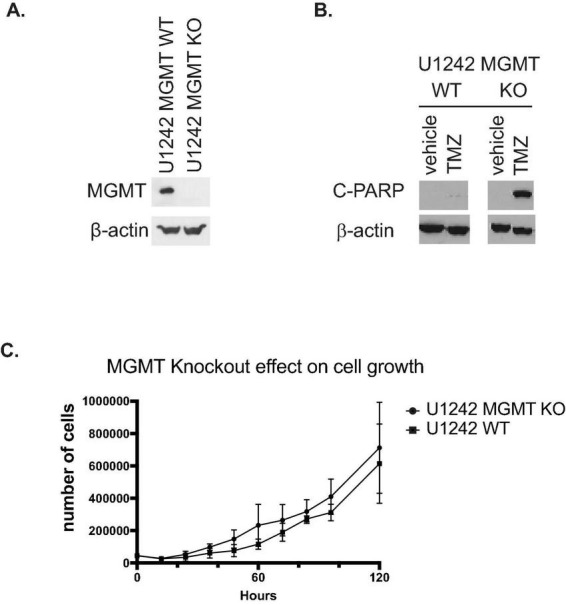
Characterization of U1210 *O*^6^-methylguanine DNA methyltransferase (MGMT) knockout (KO) cells. **(A)** Confirmation of MGMT KO in U1242 glioblastoma (GBM) cells by western blotting. **(B)** MGMT KO confers temozolomide (TMZ) sensitivity to U1242 cells. Wildtype U1242 cells are resistant to TMZ induced apoptosis as indicated by minimal detection of cleaved PARP (C PARP). MGMT KO U1242 cells undergo substantially more apoptosis after TMZ treatment. Cells were treated with 20 μM TMZ for 72 h and subjected to western blotting. **(C)** Wildtype and MGMT KO U1242 cells exhibit nearly identical proliferation rates. Growth curves of MGMT WT and MGMT KO cells were performed *in vitro*.

MGMT KO in U1242 cells was confirmed by western blotting (Figure 1A). Wildtype U1242 cells express high levels of MGMT (Sak) and are resistant to TMZ. MGMT knockout resulted in establishment of TMZ sensitivity in U1242 cells as demonstrated by elevated cleaved PARP, a marker of apoptosis (Figure 1B). Because in addition to alkylating agent treatment chemotherapy treated GBM patients, low MGMT is also associated with longer survival in radiation treated GBM patients and in some studies GBM patients independent of treatment (Hegi et al., 2005) we compared basic neoplasia-associated biological properties of MGMT KO/KD and MGMT wildtype GBM cells. We first asked the question whether MGMT KO affected the proliferation rate of U1242 cells. Standard growth curve analysis demonstrated no significant difference in the proliferation rates of wildtype and MGMT KO U1242 cells (Figure 1C).

**FIGURE 2:**
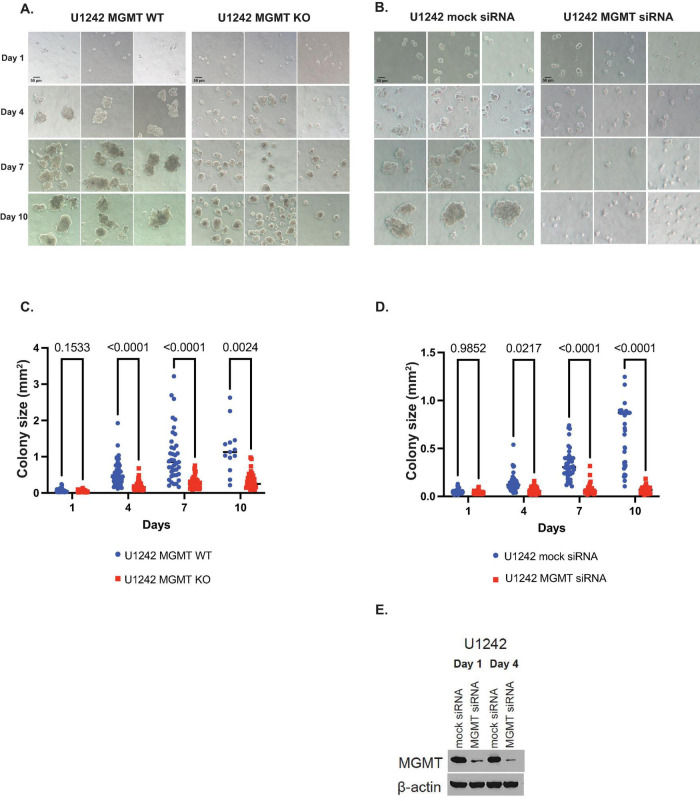
The effect of *O*^6^-methylguanine DNA methyltransferase (MGMT) expression on 3D growth of U1242 cells. **(A)** Representative photomicrographs of U1242 MGMT wildtype (WT) and knockout (KO) cell growth in soft agar on days 1, 4, 7, and 10 (scale bars represent 50 μm). **(B)** Representative photomicrographs of U1242 MGMT WT cells, transfected with mock or MGMT siRNA, in soft agar on days 1, 4, 7, and 10 (scale bars represent 50 μm). **(C)** Quantification of colony sizes of MGMT WT and KO cells. **(D)** Quantification of mock or MGMT siRNA colony sizes transfected U1242 MGMT WT cells. **(E)** Confirmation of MGMT knockdown on days 1 and 4 in U1242 cells.

To test the effect of MGMT KO on 3-dimentional anchorage-independent growth we used soft agar growth assays. MGMT KO cells showed significantly smaller cell colony spheres compared to MGMT wildtype cells on days 4, 7, and 10 of growth (*p* < 0.0001, *p* < 0.0001, and *p* = 0.0024, respectively) (Figures 2A, C). Growth of U1242 MGMT wildtype cells transfected with anti-MGMT siRNA or pooled non-targeting siRNA was also analyzed. Like KO, MGMT knockdown resulted in smaller sphere formation on days 4, 7, and 10 (*p* = 0.0217, *p* < 0.0001, and *p* < 0.0001, respectively) (Figures 2B, D). MGMT knockdown on days 1 and 4 was confirmed by western blotting (Figure 2E).

We next tested the three-dimensional growth of T98 and LN18 glioma cells, which are additional high MGMT-expressing GBM cell lines (Sak et al., 2019) transfected with anti-MGMT siRNA or non-targeting siRNA (Supplementary Figure 1). In both cell lines the cell colony spheres were consistently significantly smaller on days 7 and 10 (*p* < 000.1) when MGMT was knocked down (Supplementary Figures 1A–D). MGMT knockdown on days 1 and 4 was again confirmed by western blotting (Supplementary Figures 1E, F). Thus, MGMT KO in U1242 cells and MGMT KD in U1242, T98, and LN18 gliomas cells all decrease anchorage-independent clonogenicity in soft agar.

### MGMT knockout prevents tumor cell engraftment and growth in an orthotopic human xenograft GBM mouse model

**FIGURE 3:**
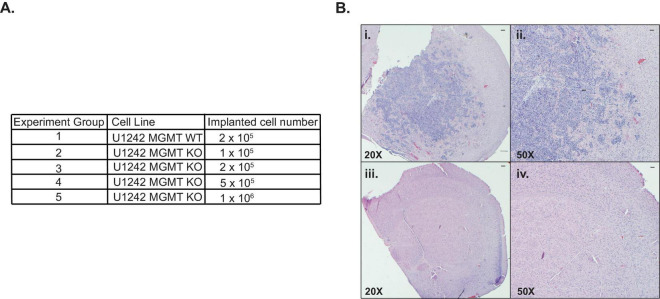
Comparison of *in vivo* growth of U1242 *O*^6^-methylguanine DNA methyltransferase (MGMT) wildtype (WT) and knockout (KO) cells. **(A)** Cell lines and implanted cell numbers for each experimental group. **(B)** Representative H&E staining of mouse brains; (i): MGMT WT 20X, (ii): MGMT WT 50X, (iii): MGMT KO 20X, (iv): MGMT KO 50X (*n* = 3 for each group, scale bars represent 200 μm).

To compare MGMT wildtype and KO U1242 tumor cell growth in mice brains, we surgically implanted an optimized amount of MGMT WT cells and four different concentrations of MGMT KO cells (Figure 3A). Animals that were implanted with MGMT WT cells showed a median survival of 54 days. However, none of the mice implanted with MGMT KO cells showed any symptoms of a tumor, such as weight loss and hunching. Mice that were implanted with MGMT KO cells were euthanized at day 70 and H&E staining revealed that no animals implanted with MGMT KO cells at any concentration formed tumors *in vivo* (Figure 3B). Even though MGMT WT and KO cells did not show any difference in growth rate *in vitro* (Figure 1C), MGMT KO prevented tumor development in mice brains.

### MGMT expression predicts response to alisertib + carboplatin treatment *in vitro*

**FIGURE 4:**
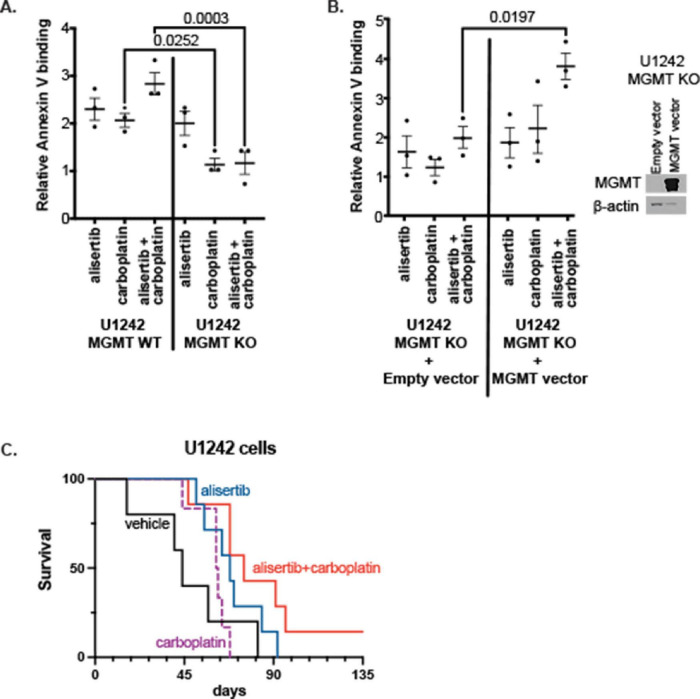
Effect of alisertib and/or carboplatin treatment on **(A)** *O*^6^-methylguanine DNA methyltransferase (MGMT) wildtype (WT) and knockout (KO) cells, and **(B)** Empty or MGMT overexpression vector transfected MGMT KO cell line (Each data point represents the mean value of three replicates from an independent experiment, *n* = 3, two-way ANOVA). **(C)** Kaplan-Meier survival curve of orthotopic xenograft U1242 MGMT WT cells implanted mice (vehicle, *n* = 5; alisertib only, *n* = 7; carboplatin only, *n* = 6; and alisertib + carboplatin, *n* = 7).

To further investigate if MGMT expression was directly involved in GBM cell response to alisertib + carboplatin treatment, we tested the effect of complete MGMT KO and exogenous MGMT replacement in response to this treatment *in vitro*. We found that wildtype MGMT-expressing cells were more sensitive to carboplatin and alisertib + carboplatin treatment compared to MGMT KO cells (*p* = 0.0252 and *p* = 0.003, respectively) (Figure 4A). MGMT overexpression in MGMT KO cells rescued the effect of alisertib + carboplatin treatment (*p* = 0.0197) (Figure 4B). Alisertib + carboplatin treatment also prolonged survival in the orthotopic xenograft mouse model; vehicle-treated mice had 48 days of median survival. Carboplatin-only treatment resulted in 60 days, and alisertib treatment alone resulted in 69 days of median animal survival. Although carboplatin itself did not show an impressive improvement, alisertib and carboplatin combination treatment extended median survival to 83 days. This improved survival was statistically significant compared to vehicle only treated animals (*p* = 0.03) (Figure 4C).

### U1242 MGMT WT and KO cells show differential DNA damage in response to alisertib and carboplatin treatment

**FIGURE 5:**
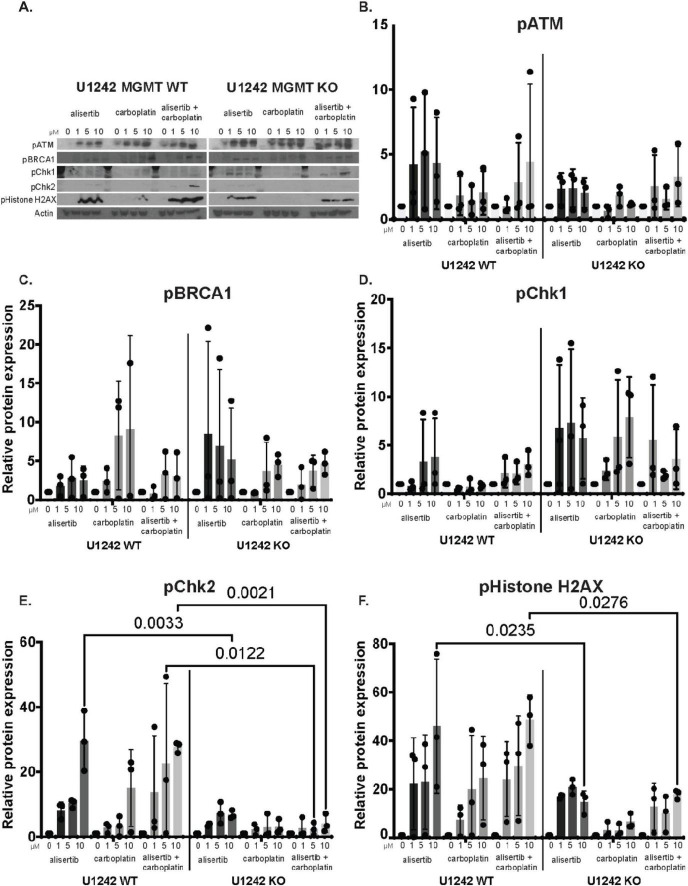
Changes in DNA damage markers in U1242 *O*^6^-methylguanine DNA methyltransferase (MGMT) wildtype (WT) and knockout (KO) cells with alisertib and carboplatin treatment. **(A)** Representative western blots of DNA damage response markers in MGMT WT and MGMT KO cells treated with 0 to 10 μM alisertib, carboplatin, or alisertib + carboplatin. Quantification of **(B)** pATM, **(C)** pBRCA1, **(D)** pChk1, **(E)**. pChk2, and **(F)** pHistone-H2AX protein levels. Each data point represents the values from independent experiments. *n* = 3, two-way ANOVA.

To compare potential differences in DNA damage response in MGMT WT and KO cells to alisertib and carboplatin treatment, we investigated pATM, pBRCA1, pChk1, pChk2, and pHistone-H2AX proteins that are involved in DNA damage pathways via western blotting (Figure 5). Although pATM appeared to be expressed at higher levels in MGMT WT cells compared to KO cells, the difference was not statistically significant (Figure 5B). pBRCA1 showed non-significant differential changes to drug treatments in both cell lines. pBRCA1 levels were higher with carboplatin treatment in MGMT WT cells compared to KO cells. In contrast, alisertib treatment resulted in higher levels of pBRCA1 in KO cells compared WT cells (Figure 5C). Even though pChk1 showed non-significant higher levels in the MGMT KO cells (Figure 5D), alisertib and alisertib + carboplatin treatment significantly increased pChk2 and pHistone-H2AX protein abundances in MGMT WT cells (Figures 5E, F). These data suggests that the MGMT WT and KO cells have different DNA repair responses to alisertib and alisertib + carboplatin treatment and the mechanism of higher efficacy of alisertib + carboplatin in high MGMT expressing WT cells may be through increased DNA damage response.

## Discussion

Current mainline chemotherapy for high-grade glioma is TMZ, which methylates guanine at the *O*^6^ position, leading to point mutations, intra-strand DNA cross-linking, double-strand breaks and apoptosis (Esteller et al., 2000; Kaina et al., 2007). TMZ may also induce senescence and downregulation of mismatch repair proteins and the homologous recombination protein RAD51 in glioma cells (Aasland et al., 2019).

In GBM, the frequent presence of high MGMT levels, which can remove the damaging *O*^6^-guanine methyl group, is associated with TMZ resistance and reduced patient survival (Iwadate et al., 2010). Reducing MGMT levels through knockdown can enhance the effectiveness of TMZ in resistant human GBM cells in animals (Viel et al., 2013) and MGMT inhibition has been shown to increase GBM cell sensitivity to TMZ (Shi et al., 2023; Kohsaka et al., 2012). However, this approach has not worked clinically using MGMT inhibitors Clinical trials involving combination therapy utilizing MGMT inhibitors and TMZ have not shown a significant increase in progression-free survival in these patients, due to toxicity (Stephen et al., 2014).

We previously found, seemingly paradoxically, that GBM cell lines expressing high MGMT levels were more sensitive to a combination of the Aurora A kinase (AURKA) inhibitor alisertib and carboplatin. Since this combination in theory does not induce guanine methylation, and because MGMT also can affect response to radiation treatment (Hegi et al., 2005), we theorized that MGMT has other biological functions unrelated to guanine methylation. We therefore knocked out MGMT using CRISPR/Cas9 in U1242 glioma cells as a potential tool to investigate possible alternate MGMT biological effects. MGMT KO did not alter the proliferation rate of these cells, however, it did substantially reduce their anchorage-independent growth and clonogenicity in soft agar. We verified this effect using siRNA KD of MGMT in parent wildtype U1242 cells and two additional high MGMT-expressing GBM cell lines, T98, and LN18. Furthermore, when we implanted the U1242 KO cells into mouse brains using a human glioma xenograft model, they failed to form tumors even at high cell inoculation concentrations.

Our previous work testing the synergy between alisertib and carboplatin in several GBM cell lines with different molecular characteristics indicated a selective synergy in high MGMT-expressing cell line (Sak et al., 2019). Our experiments consistently showed that alisertib + carboplatin treatment was more effective in MGMT-expressing cell lines, including the rescue experiment where we overexpressed MGMT in MGMT KO cells and could rescue the apoptosis caused by alisertib + carboplatin. Regardless of the mechanism of synergy, our data suggest that this combination may be effective against GBM expressing high MGMT levels, which characteristically does not respond well to chemotherapy and has a poorer prognosis.

Our *in vivo* experiments showed increased survival with the alisertib + carboplatin treatment in MGMT WT cells-implanted mice compared to vehicle-treated mice. Our attempt to test the efficacy of alisertib + carboplatin in MGMT KO cells-implanted mice was not successful since MGMT KO cells did not form tumors *in vivo*.

Carboplatin causes platinum adducts to DNA, leading to DNA crosslinking. However, it does not methylate DNA, and the connection between MGMT expression and the response to carboplatin remains uncertain. There have been conflicting reports regarding the relationship between MGMT and platinum drugs. While some studies suggest that MGMT may be capable of repairing platinum adducts (Chen et al., 2015), others purport that platinum drugs can downregulate MGMT and hasten its degradation (Tanaka et al., 2005). Nevertheless, several clinical investigations have observed positive outcomes when using platinum drugs in gliomas that express MGMT (Iwadate et al., 2010; Chen et al., 2015). Despite their distinct DNA damage mechanisms, both TMZ and carboplatin disrupt DNA replication and compromise genomic stability.

AURKA is a serine-threonine kinase that plays critical roles in cell cycle progression, mitosis, and pro-proliferative pathways (Lehman et al., 2012). AURKA is overexpressed in several neoplasms including GBM. The small molecule, selective AURKA inhibitor alisertib (MLN8237) inhibits tumor cell proliferation and causes apoptosis, differentiation, or senescence (Hong et al., 2014). Alisertib also potentiates the anti-cancer effect of many DNA-damaging agents or taxanes and the combinations are clinically used to treat several types of cancers such as breast, ovarian, and small-cell lung cancer (Falchook et al., 2019b; Falchook et al., 2019a; O’Shaughnessy et al., 2021; Owonikoko et al., 2020; Zumbar et al., 2018). We have previously demonstrated that alisertib inhibits the growth of GBM tumor stem-like cells *in vitro* and *in vivo* (Hong et al., 2014). We have tested for possible synergistic anti-glioma effects between alisertib and other treatments. We reported that alisertib potentiated the cytotoxicity of the first-line GBM adjuvant therapies temozolomide (TMZ) and ionizing radiation (Van Brocklyn et al., 2014), as well as the brain-penetrating taxane TPI 287 (Sak et al., 2023; Zumbar et al., 2018) and the cisplatin analog carboplatin (Sak et al., 2019). Carboplatin is used against a wide variety of neoplasms and is occasionally used to treat intracranial tumors in children and adults including GBM (Poisson et al., 1991; Esteller et al., 2000). Aurora Kinase A is a regulator of tumor progression and is overexpressed in GBM. We used a specific Aurora Kinase A inhibitor alisertib in combination with DNA-damaging platinum agent carboplatin. We utilized various biochemical assays, orthotopic xenograft mouse models, RNA-sequencing, and soft agar experiments to investigate the efficacy of this combination in treating *in vitro* and *in vivo* tissue culture models of GBM. Our results collectively revealed that the alisertib and carboplatin combination had selectively higher efficacy in high MGMT expressing GBM cells *in vitro* and *in vivo*.

In clinical practice, assessing tumor MGMT expression is indirectly accomplished by evaluating the methylation status of the MGMT promoter. High MGMT expression is typically associated with MGMT promoter hypomethylation, while low MGMT expression is linked to MGMT promoter hypermethylation. However, it’s important to note that this relationship does not always hold (Jung et al., 2009; Melguizo Alonso et al., 2012; Uno et al., 2011).

Although certain studies have suggested that determining MGMT promoter methylation status might be a more reliable predictor of the response to adjuvant therapy compared to directly measuring MGMT protein levels (Melguizo Alonso et al., 2012; Uno et al., 2011), there is evidence supporting the potential usefulness of *assessing* MGMT expression through immunohistochemistry in patients with unmethylated MGMT promoters but low MGMT protein expression (Melguizo Alonso et al., 2012). It is crucial to note that our study was based on MGMT protein levels, therefore consideration of this when potentially selecting patients for carboplatin-based therapies is important. In the context of such clinical studies, it is advisable to assess MGMT expression through both promoter methylation analysis and protein measurement, and then independently correlate the results with the overall response of patients.

Overall, the importance of this study is the potential effect of alisertib + carboplatin treatment in high MGMT-expressing poor prognosis GBM. While we were investigating the efficacy of this treatment, our experiments revealed a novel function of MGMT expression, specifically on *in vivo* and *in vitro* 3D growth. The effect of MGMT expression on GBM 3D cell growth was unexpected, however, it was interesting in terms of the possibility of better prognostic low-MGMT expressing tumors being less proliferative in a 3D environment in addition to being more sensitive to TMZ treatment.

We are currently conducting additional studies to help elucidate the effects of MGMT KO in tumor clonogenicity in soft agar and *in vivo*. Overall, these results show that AURKA inhibition potentiates carboplatin treatment which warrants further preclinical and clinical testing to understand the mechanisms and determine the potency in increasing GBM patient survival rate. Identification of mechanisms of action allows the stratification of patients who will benefit from these treatment options.

## Data Availability

The raw data supporting the conclusions of this article will be made available by the authors, without undue reservation.

## Footnotes

1
http://imagej.nih.gov/ijJava 1.8.0_345

## References

[B1] Aasland D. Götzinger L. Hauck L. Berte N. Meyer J. Effenberger M. (2019). Temozolomide induces senescence and repression of DNA repair pathways in glioblastoma cells via activation of ATR–CHK1, p21, and NF-κB. *Cancer Res.* 79, 99–113. 10.1158/0008-5472.CAN-18-1733 30361254

[B2] Brandes A. A. Franceschi E. Tosoni A. Blatt V. Pession A. Tallini G. (2008). MGMT promoter methylation status can predict the incidence and outcome of pseudoprogression after concomitant radiochemotherapy in newly diagnosed glioblastoma patients. *J. Clin. Oncol.* 26, 2192–2197. 10.1200/JCO.2007.14.8163 18445844

[B3] Chen S. H. Kuo C. C. Li C. F. Cheung C. H. A. Tsou T. C. Chiang H. C. (2015). O6-methylguanine DNA methyltransferase repairs platinum-DNA adducts following cisplatin treatment and predicts prognoses of nasopharyngeal carcinoma. *Int. J. Cancer* 137, 1291–1305. 10.1002/ijc.29486 25693518

[B4] Cohen A. L. Holmen S. L. Colman H. (2013). IDH1 and IDH2 mutations in gliomas. *Curr. Neurol. Neurosci. Rep*. 13, 1–7. 10.1007/s11910-013-0345-4 23532369 PMC4109985

[B5] Esteller M. Garcia-Foncillas J. Andion E. Goodman S. N. Hidalgo O. F. Vanaclocha V. (2000). Inactivation of the DNA-repair gene MGMT and the clinical response of gliomas to alkylating agents. *N. Engl. J. Med.* 343, 1350–1354. 10.1056/NEJM200011093431901 11070098

[B6] Falchook G. Coleman R. L. Roszak A. Behbakht K. Matulonis U. Ray-Coquard I. (2019a). Alisertib in combination with weekly paclitaxel in patients with advanced breast cancer or recurrent ovarian cancer: a randomized clinical trial. *JAMA Oncol*. 5:e183773. 10.1001/jamaoncol.2018.3773 30347019 PMC6439781

[B7] Falchook G. Coleman R. L. Schilder R. J. (2019b). Paclitaxel and alisertib in recurrent ovarian cancer—in reply. *JAMA Oncol.* 5, 910–911. 10.1001/jamaoncol.2019.0562 31021370

[B8] Hegi M. E. Diserens A. C. Gorlia T. Hamou M. F. De Tribolet N. Weller M. (2005). MGMT gene silencing and benefit from temozolomide in glioblastoma. *N. Engl. J. Med.* 352, 997–1003. 10.1056/NEJMoa043331 15758010

[B9] Hegi M. E. Liu L. Herman J. G. Stupp R. Wick W. Weller M. (2008). Correlation of O6-methylguanine methyltransferase (MGMT) promoter methylation with clinical outcomes in glioblastoma and clinical strategies to modulate MGMT activity. *J. Clin. Oncol.* 26, 4189–4199. 10.1200/JCO.2007.11.5964 18757334

[B10] Hong X. O’Donnell J. P. Salazar C. R. Van Brocklyn J. R. Barnett K. D. Pearl D. K. (2014). The selective Aurora-A kinase inhibitor MLN8237 (alisertib) potently inhibits proliferation of glioblastoma neurosphere tumor stem-like cells and potentiates the effects of temozolomide and ionizing radiation. *Cancer Chemother. Pharmacol.* 73, 983–990. 10.1007/s00280-014-2430-z 24627220 PMC4975936

[B11] Iwadate Y. Matsutani T. O. M. O. O. Hasegawa Y. Shinozaki N. Oide T. Tanizawa T. (2010). Selection of chemotherapy for glioblastoma expressing O6-methylguanine-DNA methyltransferase. *Exp. Ther. Med*. 1, 53–57. 10.3892/etm_00000009 23136592 PMC3490398

[B12] Jiang P. Mukthavaram R. Chao Y. Nomura N. Bharati I. S. Fogal V. (2014). *In vitro* and *in vivo* anticancer effects of mevalonate pathway modulation on human cancer cells. *Br. J. Cancer* 111, 1562–1571. 10.1038/bjc.2014.431 25093497 PMC4200085

[B13] Jung T. Y. Jung S. Jin S. G. Moon K. S. Kim I. Y. Kang S. S. (2009). The correlation and prognostic significance of MGMT promoter methylation and MGMT protein in glioblastomas. *Neurosurgery* 65, 866–875. 10.1227/01.NEU.0000357325.90347.A1 19834398

[B14] Kaina B. Christmann M. Naumann S. Roos W. P. (2007). MGMT: key node in the battle against genotoxicity, carcinogenicity and apoptosis induced by alkylating agents. *DNA Repair* 6, 1079–1099. 10.1016/j.dnarep.2007.03.008 17485253

[B15] Kohsaka S. Wang L. Yachi K. Mahabir R. Narita T. Itoh T. (2012). STAT3 inhibition overcomes temozolomide resistance in glioblastoma by downregulating MGMT expression. *Mol. Cancer Ther.* 11, 1289–1299. 10.1158/1535-7163.MCT-11-0801 22532597

[B16] Lehman N. L. O’Donnell J. P. Whiteley L. J. Stapp R. T. Lehman T. D. Roszka K. M. (2012). Aurora A is differentially expressed in gliomas, is associated with patient survival in glioblastoma and is a potential chemotherapeutic target in gliomas. *Cell Cycle* 11, 489–502. 10.4161/cc.11.3.18996 22274399 PMC3315093

[B17] Louis D. N. Aldape K. D. Capper D. Giannini C. Horbinski C. M. Ng H. K. (2021) ‘*Glioblastoma, IDH-wildtype,’ central nervous system tumors*, 5th Edn, ed. WHO classification of tumors editorial board. Lyon: International agency for research on cancer, 39–55.

[B18] Melguizo Alonso C. Prados Salazar J. C. Alvarez P. J. Perazzoli G. Oliver J. A. López R. (2012). MGMT promoter methylation status and MGMT and CD133 immunohistochemical expression as prognostic markers in glioblastoma patients treated with temozolomide plus radiotherapy. *J. Transl. Med*. 10:250. 10.1186/1479-5876-10-250 23245659 PMC3551841

[B19] Okamoto R. Takano H. Okamura T. Park J. S. Tanimoto K. Sekikawa T. (2002). O6-methylguanine-DNA methyltransferase (MGMT) as a determinant of resistance to camptothecin derivatives. *Jpn. J. Cancer Res*. 93, 93–102. 10.1111/j.1349-7006.2002.tb01205.x 11802813 PMC5926864

[B20] O’Shaughnessy J. McIntyre K. Wilks S. Ma L. Block M. Andorsky D. (2021). Efficacy and safety of weekly paclitaxel with or without oral alisertib in patients with metastatic breast cancer: a randomized clinical trial. *JAMA Netw. Open* 4:e214103. 10.1001/jamanetworkopen.2021.4103 33877311 PMC8058641

[B21] Owonikoko T. K. Niu H. Nackaerts K. Csoszi T. Ostoros G. Mark Z. (2020). Randomized phase II study of paclitaxel plus alisertib versus paclitaxel plus placebo as second-line therapy for SCLC: primary and correlative biomarker analyses. *J. Thorac. Oncol.* 15, 274–287. 10.1016/j.jtho.2019.10.013 31655296

[B22] Poisson M. Pereon Y. Chiras J. Delattre J. Y. (1991). Treatment of recurrent malignant supratentorial gliomas with carboplatin (CBDCA). *J. Neurooncol.* 10, 139–144. 10.1007/BF00146875 1654401

[B23] Sak M. Williams B. J. Zumbar C. T. Teer L. Al-Kawaaz M. N. Kakar A. (2023). The CNS-penetrating taxane drug TPI 287 potentiates antiglioma activity of the AURKA inhibitor alisertib *in vivo*. *Cancer Chemother. Pharmacol*. 91, 191–201. 10.1007/s00280-023-04503-0 36694044

[B24] Sak M. Zumbar C. T. King P. D. Li X. Mifsud C. S. Usubalieva A. (2019). Cytotoxic synergy between alisertib and carboplatin versus alisertib and irinotecan are inversely dependent on MGMT levels in glioblastoma cells. *J. Neurooncol.* 143, 231–240. 10.1007/s11060-019-03164-5 31011934 PMC6727196

[B25] Shi J. Zhang P. Dong X. Yuan J. Li Y. Li S. (2023). METTL3 knockdown promotes temozolomide sensitivity of glioma stem cells via decreasing MGMT and APNG mRNA stability. *Cell Death Discov.* 9:22. 10.1038/s41420-023-01327-y 36683086 PMC9868123

[B26] Stephen Z. R. Kievit F. M. Veiseh O. Chiarelli P. A. Fang C. Wang K. (2014). Redox-responsive magnetic nanoparticle for targeted convection-enhanced delivery of O6-benzylguanine to brain tumors. *ACS Nano* 8, 10383–10395. 10.1021/nn503735w 25247850 PMC4212796

[B27] Stoyanov G. S. Lyutfi E. Georgieva R. Georgiev R. Dzhenkov D. L. Petkova L. (2022). Reclassification of glioblastoma multiforme according to the 2021 World Health Organization classification of central nervous system tumors: a single institution report and practical significance. *Cureus* 14:e21822. 10.7759/cureus.21822 35291535 PMC8896839

[B28] Tanaka S. Kobayashi I. Utsuki S. Oka H. Yasui Y. Fujii K. (2005). Down-regulation of O6-methylguanine- DNA methyltransferase gene expression in gliomas by platinum compounds. *Oncol. Rep.* 14, 1275–1280. 10.3892/or.14.5.1275 16211296

[B29] Uno M. Oba-Shinjo S. M. Camargo A. A. Moura R. P. de Aguiar P. H. Cabrera H. N. (2011). Correlation of MGMT promoter methylation status with gene and protein expression levels in glioblastoma. *Clinics* 66, 1747–1755. 10.1590/S1807-59322011001000013 22012047 PMC3180167

[B30] Van Brocklyn J. R. Wojton J. Meisen W. H. Kellough D. A. Ecsedy J. A. Kaur B. (2014). Aurora-A inhibition offers a novel therapy effective against intracranial glioblastoma. *Cancer Res.* 74, 5364–5370. 10.1158/0008-5472.CAN-14-0386 25106428 PMC4528677

[B31] Viel T. Monfared P. Schelhaas S. Fricke I. B. Kuhlmann M. T. Fraefel C. (2013). Optimizing glioblastoma temozolomide chemotherapy employing lentiviral-based anti-MGMT shRNA technology. *Mol. Ther.* 21, 570–579. 10.1038/mt.2012.278 23319055 PMC3589165

[B32] Weller M. Stupp R. Reifenberger G. Brandes A. A. Van Den Bent M. J. Wick W. (2010). MGMT promoter methylation in malignant gliomas: ready for personalized medicine? *Nat. Rev. Neurol.* 6, 39–51. 10.1038/nrneurol.2009.197 19997073

[B33] Westermark B. Pontén J. Hugosson R. (1973). Determinants for the establishment of permanent tissue culture lines from human gliomas. *Acta Pathol. Microbiol. Scand. A Pathol.* 81, 791–805. 10.1111/j.1699-0463.1973.tb03573.x 4359449

[B34] Zhao Y. Xiao A. Carpenter J. E. Abdel-Fattah R. Redpath G. T. Lopes M. B. S. (2010). An extensive invasive intracranial human glioblastoma xenograft model: role of high level matrix metalloproteinase 9. *Am. J. Pathol.* 176, 3032–3049. 10.2353/ajpath.2010.090571 20413683 PMC2877863

[B35] Zumbar C. T. Usubalieva A. King P. D. Li X. Mifsud C. S. Dalton H. M. (2018). The CNS penetrating taxane TPI 287 and the AURKA inhibitor alisertib induce synergistic apoptosis in glioblastoma cells. *J. Neurooncol.* 137, 481–492. 10.1007/s11060-018-2755-2 29396807 PMC6097628

