# Corrigendum: *O*^6^-methylguanine DNA methyltransferase (MGMT) expression in U1242 glioblastoma cells enhances *in vitro* clonogenicity, tumor implantation *in vivo*, and *sensitivity* to alisertib-carboplatin combination treatment

**DOI:** 10.3389/fncel.2025.1637837

**Published:** 2025-06-23

**Authors:** Müge Sak, Brian J. Williams, Andrew J. Hey, Mayur Sharma, Leslie Schier, Megan J. Wilson, Mahatma Ortega, Alyssa I. Lara, Mikaela N. Brentlinger, Norman L. Lehman

**Affiliations:** ^1^Departments of Biochemistry and Molecular Genetics, University of Louisville, Louisville, KY, United States; ^2^Pathology and Laboratory Medicine, University of Louisville, Louisville, KY, United States; ^3^Neurological Surgery, University of Louisville, Louisville, KY, United States; ^4^Brown Cancer Center, University of Louisville, Louisville, KY, United States; ^5^Departments of Pathology and Laboratory Medicine, Baylor Scott & White Health, Baylor College of Medicine, Temple, TX, United States; ^6^Department of Pathology and Immunology, Baylor College of Medicine, Houston, TX, United States

**Keywords:** Aurora A Kinase, AURKA, *O*^6^-methylguanine DNA methyltransferase, MGMT, GBM, anchorage-independent growth, clonogenicity, *in vivo*

In the published article, there was an error in the article **title**. Instead of “O 6 -methylguanine DNA methyltransferase (MGMT) expression in U1242 GBM cells enhances *in vitro* clonogenicity, tumor implantation *in vivo*, and sensitivity to alisertibcarboplatin combination treatment”, it should be “*O*^6^*-methylguanine DNA methyltransferase (MGMT) expression in U1242 glioblastoma cells enhances in vitro clonogenicity, tumor implantation in vivo, and sensitivity to alisertib-carboplatin combination treatment.”*

In the published article, the **references** for all of the citations was incorrectly written. It should be:

Aasland, D., Götzinger, L., Hauck, L., Berte, N., Meyer, J., Effenberger, M., et al. (2019). Temozolomide induces senescence and repression of DNA repair pathways in glioblastoma cells via activation of ATR–CHK1, p21, and NF-κB. *Cancer Res*. 79, 99–113. 10.1158/0008-5472.CAN-18-1733

Brandes, A. A., Franceschi, E., Tosoni, A., Blatt, V., Pession, A., Tallini, G., et al. (2008). MGMT promoter methylation status can predict the incidence and outcome of pseudoprogression after concomitant radiochemotherapy in newly diagnosed glioblastoma patients. *J. Clin. Oncol*. 26, 2192–2197.doi: 10.1200/JCO.2007.14.8163

Jung, T. Y., Jung, S., Jin, S. G., Moon, K. S., Kim, I. Y., Kang, S. S., et al. (2009). The correlation and prognostic significance of MGMT promoter methylation and MGMT protein in glioblastomas. *Neurosurgery* 65, 866–875.doi: 10.1227/01.NEU.0000357325.90347.A1

Chen, S. H., Kuo, C. C., Li, C. F., Cheung, C. H. A., Tsou, T. C., Chiang, H. C., et al. (2015). O6-methylguanine DNA methyltransferase repairs platinum-DNA adducts following cisplatin treatment and predicts prognoses of nasopharyngeal carcinoma. *Int. J. Cancer* 137, 1291–1305.doi: 10.1002/ijc.29486

Cohen, A. L., Holmen, S. L., and Colman, H. (2013). IDH1 and IDH2 mutations in gliomas. *Curr. Neurol. Neurosci. Rep*. 13, 1–7.doi: 10.1007/s11910-013-0345-4

Esteller, M., Garcia-Foncillas, J., Andion, E., Goodman, S. N., Hidalgo, O. F., Vanaclocha, V., et al. (2000). Inactivation of the DNA-repair gene MGMT and the clinical response of gliomas to alkylating agents. *N. Engl. J. Med*. 343, 1350–1354.doi: 10.1056/NEJM200011093431901

Falchook, G., Coleman, R. L., Roszak, A., Behbakht, K., Matulonis, U., Ray-Coquard, I., et al. (2019). Alisertib in combination with weekly paclitaxel in patients with advanced breast cancer or recurrent ovarian cancer: a randomized clinical trial. *JAMA Oncol*. 5:e183773.doi: 10.1001/jamaoncol.2018.3773

Falchook, G., Coleman, R. L., and Schilder, R. J. (2019). Paclitaxel and alisertib in recurrent ovarian cancer—in reply. *JAMA Oncol*. 5, 910–911.doi: 10.1001/jamaoncol.2019.0562

Hegi, M. E., Diserens, A. C., Gorlia, T., Hamou, M. F., De Tribolet, N., Weller, M., et al. (2005). MGMT gene silencing and benefit from temozolomide in glioblastoma. *N. Engl. J. Med*. 352, 997–1003.doi: 10.1056/NEJMoa043331

Hegi, M. E., Liu, L., Herman, J. G., Stupp, R., Wick, W., Weller, M., et al. (2008). Correlation of O6-methylguanine methyltransferase (MGMT) promoter methylation with clinical outcomes in glioblastoma and clinical strategies to modulate MGMT activity. *J. Clin. Oncol*. 26, 4189–4199.doi: 10.1200/JCO.2007.11.5964

Hong, X., O'Donnell, J. P., Salazar, C. R., Van Brocklyn, J. R., Barnett, K. D., Pearl, D. K., et al. (2014). The selective Aurora-A kinase inhibitor MLN8237 (alisertib) potently inhibits proliferation of glioblastoma neurosphere tumor stem-like cells and potentiates the effects of temozolomide and ionizing radiation. *Cancer Chemother. Pharmacol*. 73, 983–990.doi: 10.1007/s00280-014-2430-z

Iwadate, Y., Matsutani, T. O. M. O. O., Hasegawa, Y., Shinozaki, N., Oide, T., Tanizawa, T., et al. (2010). Selection of chemotherapy for glioblastoma expressing O 6-methylguanine-DNA methyltransferase. *Exp. Ther. Med*. 1, 53–57.doi: 10.3892/etm_00000009

Jiang, P., Mukthavaram, R., Chao, Y., Nomura, N., Bharati, I. S., Fogal, V., et al. (2014). *In vitro* and *in vivo* anticancer effects of mevalonate pathway modulation on human cancer cells. *Br. J. Cancer* 111, 1562–1571.doi: 10.1038/bjc.2014.431

Kaina, B., Christmann, M., Naumann, S., and Roos, W. P. (2007). MGMT: key node in the battle against genotoxicity, carcinogenicity and apoptosis induced by alkylating agents. *DNA Repair* 6, 1079–1099.doi: 10.1016/j.dnarep.2007.03.008

Kohsaka, S., Wang, L., Yachi, K., Mahabir, R., Narita, T., Itoh, T., et al. (2012). STAT3 inhibition overcomes temozolomide resistance in glioblastoma by downregulating MGMT expression. *Mol. Cancer Ther*. 11, 1289–1299.doi: 10.1158/1535-7163.MCT-11-0801

Lehman, N. L., O'Donnell, J. P., Whiteley, L. J., Stapp, R. T., Lehman, T. D., Roszka, K. M., et al. (2012). Aurora A is differentially expressed in gliomas, is associated with patient survival in glioblastoma and is a potential chemotherapeutic target in gliomas. *Cell Cycle* 11, 489–502.doi: 10.4161/cc.11.3.18996

Louis, D. N., Aldape, K. D., Capper, D., Giannini, C., Horbinski, C. M., Ng, H. K., et al. (2021). ‘*Glioblastoma, IDH-Wildtype', Central Nervous System Tumors, Edited by WHO Classification of Tumors Editorial Board, 5th Edition*. Lyon: International agency for research on cancer, 39–55.

Melguizo Alonso, C., Prados Salazar, J. C., Alvarez, P. J., Perazzoli, G., Oliver, J. A., López, R., et al. (2012). MGMT promoter methylation status and MGMT and CD133 immunohistochemical expression as prognostic markers in glioblastoma patients treated with temozolomide plus radiotherapy. *J. Transl. Med*. 10:250.doi: 10.1186/1479-5876-10-250

Okamoto, R., Takano, H., Okamura, T., Park, J. S., Tanimoto, K., Sekikawa, T., et al. (2002). O6-methylguanine-DNA methyltransferase (MGMT) as a determinant of resistance to camptothecin derivatives. *Jpn J. Cancer Res*. 93, 93–102.doi: 10.1111/j.1349-7006.2002.tb01205.x

O'Shaughnessy, J., McIntyre, K., Wilks, S., Ma, L., Block, M., Andorsky, D., et al. (2021). Efficacy and safety of weekly paclitaxel with or without oral alisertib in patients with metastatic breast cancer: a randomized clinical trial. *JAMA Netw. Open* 4:e214103.doi: 10.1001/jamanetworkopen.2021.4103

Owonikoko, T. K., Niu, H., Nackaerts, K., Csoszi, T., Ostoros, G., Mark, Z., et al. (2020). Randomized phase II study of paclitaxel plus alisertib versus paclitaxel plus placebo as second-line therapy for SCLC: primary and correlative biomarker analyses. *J. Thorac. Oncol*. 15, 274–287.doi: 10.1016/j.jtho.2019.10.013

Poisson, M., Pereon, Y., Chiras, J., and Delattre, J. Y. (1991). Treatment of recurrent malignant supratentorial gliomas with carboplatin (CBDCA). *J. Neurooncol*. 10, 139–144.doi: 10.1007/BF00146875

Sak, M., Zumbar, C. T., King, P. D., Li, X., Mifsud, C. S., Usubalieva, A., et al. (2019). Cytotoxic synergy between alisertib and carboplatin versus alisertib and irinotecan are inversely dependent on MGMT levels in glioblastoma cells. *J. Neurooncol*. 143, 231–240.doi: 10.1007/s11060-019-03164-5

Sak, M., Williams, B. J., Zumbar, C. T., Teer, L., Al-Kawaaz, M. N., Kakar, A., et al. (2023). The CNS-penetrating taxane drug TPI 287 potentiates antiglioma activity of the AURKA inhibitor alisertib *in vivo*. *Cancer Chemother. Pharmacol*. 91, 191–201.doi: 10.1007/s00280-023-04503-0

Stoyanov, G. S., Lyutfi, E., Georgieva, R., Georgiev, R., Dzhenkov, D. L., Petkova, L., et al. (2022). Reclassification of glioblastoma multiforme according to the 2021 World Health Organization classification of central nervous system tumors: a single institution report and practical significance. *Cureus* 14:e21822.doi: 10.7759/cureus.21822

Shi, J., Zhang, P., Dong, X., Yuan, J., Li, Y., Li, S., et al. (2023). METTL3 knockdown promotes temozolomide sensitivity of glioma stem cells via decreasing MGMT and APNG mRNA stability. *Cell Death Discov*. 9:22.doi: 10.1038/s41420-023-01327-y

Stephen, Z. R., Kievit, F. M., Veiseh, O., Chiarelli, P. A., Fang, C., Wang, K., et al. (2014). Redox-responsive magnetic nanoparticle for targeted convection-enhanced delivery of O 6-benzylguanine to brain tumors. *ACS Nano* 8, 10383–10395.doi: 10.1021/nn503735w

Tanaka, S., Kobayashi, I., Utsuki, S., Oka, H., Yasui, Y., Fujii, K., et al. (2005). Down-regulation of O6-methylguanine-DNA methyltransferase gene expression in gliomas by platinum compounds. *Oncol. Rep*. 14, 1275–1280.doi: 10.3892/or.14.5.1275

Uno, M., Oba-Shinjo, S. M., Camargo, A. A., Moura, R. P., de Aguiar, P. H., Cabrera, H. N., et al. (2011). Correlation of MGMT promoter methylation status with gene and protein expression levels in glioblastoma. *Clinics* 66, 1747–1755.doi: 10.1590/S1807-59322011001000013

Van Brocklyn, J. R., Wojton, J., Meisen, W. H., Kellough, D. A., Ecsedy, J. A., Kaur, B., et al. (2014). Aurora-A inhibition offers a novel therapy effective against intracranial glioblastoma. *Cancer Res*. 74, 5364–5370.doi: 10.1158/0008-5472.CAN-14-0386

Viel, T., Monfared, P., Schelhaas, S., Fricke, I. B., Kuhlmann, M. T., Fraefel, C., et al. (2013). Optimizing glioblastoma temozolomide chemotherapy employing lentiviral-based anti-MGMT shRNA technology. *Mol. Ther*. 21, 570–579.doi: 10.1038/mt.2012.278

Westermark, B., Ponten, J., and Hugosson, R. (1973). Determinants for the establishment of permanent tissue culture lines from human gliomas. *Acta Pathol. Microbiol. Scand. A Pathol*. 81, 791–805.doi: 10.1111/j.1699-0463.1973.tb03573.x

Weller, M., Stupp, R., Reifenberger, G., Brandes, A. A., Van Den Bent, M. J., Wick, W., et al. (2010). MGMT promoter methylation in malignant gliomas: ready for personalized medicine? *Nat. Rev. Neurol*. 6, 39–51.doi: 10.1038/nrneurol.2009.197

Zhao, Y., Xiao, A., Carpenter, J. E., Abdel-Fattah, R., Redpath, G. T., Lopes, M. B. S., et al. (2010). An extensive invasive intracranial human glioblastoma xenograft model: role of high level matrix metalloproteinase 9. *Am J. Pathol*. 176, 3032–3049.doi: 10.2353/ajpath.2010.090571

Zumbar, C. T., Usubalieva, A., King, P. D., Li, X., Mifsud, C. S., Dalton, H. M., et al. (2018). The CNS penetrating taxane TPI 287 and the AURKA inhibitor alisertib induce synergistic apoptosis in glioblastoma cells. *J. Neurooncol*. 137, 481–492.doi: 10.1007/s11060-018-2755-2

In the published article, there was an error in **Results**, paragraph 5. The sentence previously stated:

“Thus MGMT KO in U1210 cells and MGMT KD in U1210, T98 and LN18 gliomas cells all decrease anchorage-independent clonogenicity in soft agar.”

The corrected sentence appears below:

“Thus, MGMT KO in U1242 cells and MGMT KD in U1242, T98, and LN18 gliomas cells all decrease anchorage-independent clonogenicity in soft agar.”

In the published article, affiliation 6 was erroneously omitted and the corresponding affiliation number was not added for author Norman L. Lehman. Affiliation 6 has now been added as below: “6. Department of Pathology and Immunology, Baylor College of Medicine, Houston, TX, United States”

The authors apologize for these errors and state that they do not change the scientific conclusions of the article in anyway. The original article has been updated.

