# Supplementary figures and images for: O6-methylguanine DNA methyltransferase (MGMT) expression in U1242 glioblastoma cells enhances in vitro clonogenicity, tumor implantation in vivo, and sensitivity to alisertib-carboplatin combination treatment
Source: Front Cell Neurosci. 2025 Apr 22;19:1552015. doi: 10.3389/fncel.2025.1552015 (PMC12056744; doi:10.3389/fncel.2025.1552015)

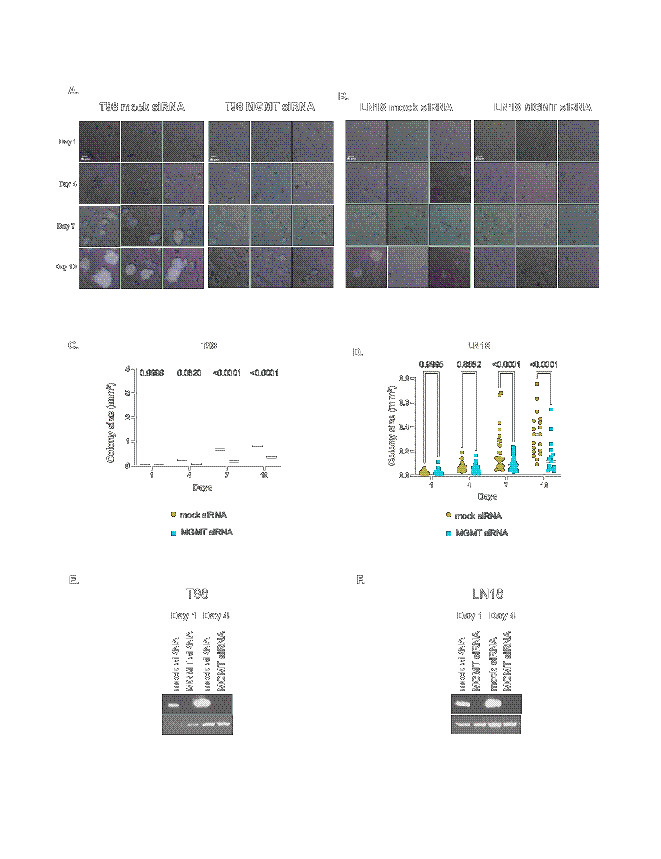

Supplement: Supplementary file 1 [file Image_1.jpg]
